# Neutrophil-to-Lymphocyte Ratio: A Biomarker to Monitor the Immune Status of Astronauts

**DOI:** 10.3389/fimmu.2020.564950

**Published:** 2020-11-02

**Authors:** Amber M. Paul, Siddhita D. Mhatre, Egle Cekanaviciute, Ann-Sofie Schreurs, Candice G. T. Tahimic, Ruth K. Globus, Sulekha Anand, Brian E. Crucian, Sharmila Bhattacharya

**Affiliations:** ^1^ Space Biosciences Division, NASA Ames Research Center, Moffett Field, CA, United States; ^2^ Universities Space Research Association, Columbia, MD, United States; ^3^ COSMIAC Research Center, University of New Mexico, Albuquerque, NM, United States; ^4^ KBR, Houston, TX, United States; ^5^ Department of Biology, University of North Florida, Jacksonville, FL, United States; ^6^ Department of Biological Sciences, San Jose State University, San Jose, CA, United States; ^7^ Biomedical Research and Environmental Sciences Division, NASA Johnson Science Center, Houston, TX, United States

**Keywords:** neutrophils, spaceflight, simulated microgravity, NLR, inflammation, oxidative stress response

## Abstract

A comprehensive understanding of spaceflight factors involved in immune dysfunction and the evaluation of biomarkers to assess in-flight astronaut health are essential goals for NASA. An elevated neutrophil-to-lymphocyte ratio (NLR) is a potential biomarker candidate, as leukocyte differentials are altered during spaceflight. In the reduced gravity environment of space, rodents and astronauts displayed elevated NLR and granulocyte-to-lymphocyte ratios (GLR), respectively. To simulate microgravity using two well-established ground-based models, we cultured human whole blood-leukocytes in high-aspect rotating wall vessels (HARV-RWV) and used hindlimb unloaded (HU) mice. Both HARV-RWV simulation of leukocytes and HU-exposed mice showed elevated NLR profiles comparable to spaceflight exposed samples. To assess mechanisms involved, we found the simulated microgravity HARV-RWV model resulted in an imbalance of redox processes and activation of myeloperoxidase-producing inflammatory neutrophils, while antioxidant treatment reversed these effects. In the simulated microgravity HU model, mitochondrial catalase-transgenic mice that have reduced oxidative stress responses showed reduced neutrophil counts, NLR, and a dampened release of selective inflammatory cytokines compared to wildtype HU mice, suggesting simulated microgravity induced oxidative stress responses that triggered inflammation. In brief, both spaceflight and simulated microgravity models caused elevated NLR, indicating this as a potential biomarker for future in-flight immune health monitoring.

## Introduction

Spaceflight can pose novel challenges to the health of astronauts. For instance, physiological aging occurs significantly faster as a result of spaceflight, when measured by muscle wasting, loss of bone density, and immune dysfunction (1, 2). Processes regulated by redox imbalance may contribute to these adverse outcomes (3–9). Redox imbalance results from a disproportionate increase in reactive oxygen species (ROS) produced by the mitochondria (10) compared to antioxidants in the cell. Elevated ROS is also a product of the oxidative burst response of neutrophils (11). In response to stimuli, terminally differentiated neutrophils in circulation become activated and engage the oxidative burst response, producing inflammatory mediators (11). If left unchecked, elevated ROS can cause cellular damage that potentiates inflammation both on Earth and during spaceflight (4, 12). Therefore, it is necessary to maintain tight regulation of the oxidative burst response to limit inflammation (13) and regulate immunity during prolonged spaceflight.

Neutrophils are granulocytes that constitute approximately 50–70% of the total leukocyte population in humans. Neutrophils are the first responders to infection or injury and are typically short-lived in blood circulation under homeostatic conditions (14, 15). Lymphocytes are an important group of white blood cells involved in both innate and adaptive immunity. They constitute 20–50% of total leukocytes in circulation and consist of natural killer, natural killer T cells, innate lymphoid cells, T cells, and B cells (16, 17). On Earth, elevated neutrophil-to-lymphocyte ratio (NLR) is a useful biomarker to measure subclinical inflammation in humans (18). Chronic, persistent inflammation can be a major pre-existing cause of disease development (19, 20) and can be monitored by the expression of blood-based biomarkers. For example, elevated NLR predicts poor prognosis in some cancers (21–24), positively correlates with age (25), and reflects chronic stress in mice (26). Although, elevated human NLR (>3.53) (27) has been implicated in clinical settings to identify heightened inflammation (18), this biomarker has not yet been recognized for spaceflight-induced inflammation. Spaceflight raises circulating white blood cell (WBC) counts, primarily granulocytes, may reduce lymphocyte counts (12, 28, 29), and impairs immune cell functions (6, 12, 30, 31). Although, the spaceflight environment elevates circulating blood granulocytes counts in astronauts (29), the underlying molecular mechanisms remain elusive. Currently, there are no well-established biomarkers for astronauts on long-duration, deep space missions, where medical intervention will be limited. Thus, identifying biomarkers to monitor in-flight astronaut health and developing countermeasures that reverse these adverse outcomes are necessary for successful future missions to the lunar surface and Mars. Therefore, we propose that an elevated NLR may be a useful prognostic indicator or diagnostic biomarker to assess astronaut immune status during long-duration missions.

To test this, we analyzed both spaceflight-treated, and ground-based simulated microgravity-treated, samples to determine if NLR was elevated. Analyses of complete blood count (CBC) leukocyte differentials revealed spaceflight caused a progressive increase of granulocyte-to-lymphocyte (GLR) in astronauts and NLR in rodents. To simulate microgravity using established methods, human leukocytes were cultured in high-aspect rotating wall vessels (HARV-RWV) *in vitro* and mice were hindlimb unloaded (HU) *in vivo* (32, 33). HARV-RWV is a bioreactor allowing 3D-spatial freedom for cells and can model microgravity. It has two unique aspects similar to the spaceflight-associated microgravity environment, (1) a state of constant suspension, and (2) a quiescent surrounding without any shear or turbulent forces. Previous studies have determined leukocyte responses utilizing HARV-RWV produce similar responses as leukocytes cultured post-landing or *ex vivo* in flight (34–42). Hindlimb unloading (HU) is a ground-based model mimicking spaceflight-associated microgravity in rodents. The hindlimbs of rodents are elevated to produce 30–40 degree head-down tilt, inducing a cephalad fluid shift and preventing weightbearing of hindlimbs (43). The HU model can lead to immune, bone, and musculoskeletal alterations, some of which have also been observed in International Space Station (ISS) crew (44, 45). In our study, functional outputs of neutrophils in response to simulated microgravity (sµg) revealed elevated ROS and proinflammatory myeloperoxidase (MPO) expression in activated neutrophils. Interestingly, this effect could be mitigated with antioxidant treatment. Furthermore, sµg HU *wildtype* (*Wt*) mice displayed elevated neutrophils, NLR and marginal inflammation, which was dampened in mitochondrial catalase (*mCAT*) transgenic mice, known to show reduced oxidative stress responses. Our findings demonstrated that, albeit distinct mechanisms, both sµg models (*in vitro* HARV-RWV and *in vivo* HU), displayed elevated oxidative stress and NLR, that could be mitigated by antioxidants. Therefore, modifying mechanisms involved in ROS-driven inflammation (46) may provide a promising avenue to limit chronic inflammation and maintain homeostatic immunity during long-duration missions.

## Materials and Methods

### Mouse and Human Ethics

Deidentified, human buffy coat samples from healthy donors were obtained from Blood Centers of America, Oklahoma Blood Institute, and isolated on-site at NASA Ames. The use of human samples was approved by NASA Ames Institutional Review Board (IRB, 201791646CTO-02, HR-357, and HR-358) with informed consent from each blood donor. Astronaut and rodent CBC data sets were approved for use by the electronic (e)IRB/Life Sciences Data Archive (LSDA) advisory board (#11028), sourced from previous publications (29, 47, 48). All mice were purchased from Jackson Laboratories and were housed in the Animal Care Facility at Ames Research Center. Hindlimb unloading and subsequent blood isolation procedures were performed following NASA Ames Research Center Institutional Animal Care and Use Committee protocol (IACUC, NAS-17-001-Y2).

### Cell Culture

Human whole blood samples were separated using centrifuge gradient Ficoll-paque Plus (Thermo Fisher Scientific) and the lymphocyte/monocyte layer and granulocyte/top red blood cell (RBC) layers were collected. Cells were RBC-lysed with 1XRBC lysis buffer (Thermo Fisher Scientific) and resuspended (5 × 10^5^ cells/ml) in RPMI containing 10% fetal bovine serum (FBS) and 1% Penicillin/Streptomycin (Pen/Strep, Thermo Fisher Scientific) for subsequent assays.

### *In Vitro* Simulated Microgravity of Leukocytes Using HARV-RWV

3D high-aspect rotating wall vessels (HARV-RWV, Synthecon) were used to simulate microgravity with low-shear, hydrodynamic fluid flow and omni-directional gravitational force on suspended cells in rotating free-fall (34, 49–51). To optimize the measurement of oxidative stress from granulocytes in human leukocytes, suspended cells were cultured at 5 × 10^5^ cells/ml in 10 ml and rotated at 20 revolutions per minute (RPM) in a parallel-to-ground axis to simulate microgravity (sµg, omnidirectional g-force) for 20 h and controls were plated in upright T-25 flasks (1 g, unidirectional g-force). Following incubation, 1 ml of sample was collected, SYTOX™ live/dead dye-Red (Thermo Fisher Scientific) was added to the sample and cells were immediately acquired on a BD FACSMelody™. Stained cells were considered dead, and cells that did not stain were considered live and were reported. For neutrophil differential experiments with antioxidant treatment, *N*-acetyl cysteine (NAC, 1 mM) was added to WBC (5 × 10^5^ cells/ml) and cultured in HARV-RWV for 20 h, followed by flow cytometric analyses.

### Mouse Blood Collections

Blood was collected from the vena cava on the day of euthanasia and RBC were lysed using 1XRBC lysis buffer (Thermo Fisher Scientific). Remaining WBC were fixed (2% PFA), washed in PBS, labeled with leukocyte subset markers, and analyzed by flow cytometric analyses.

### Flow Cytometry Staining and Methods

Mouse and human blood samples were isolated, as described above, and single-cell suspensions were generated for flow cytometry acquisition. Debris was gated off and forward scatter (FSC-A) and side scatter (SSC-A) profiled granulocyte, monocyte, and lymphocyte populations were measured. Mouse antibodies, including anti-CD45, anti-Ly6g, and anti-CD11b, and human antibodies, including anti-CD66b, anti-CD16, anti-MPO, CellROX™, SYTOX™ live/dead stain, and active Caspase 3/7 were used to label multiple leukocyte subsets, and measure ROS formation and cellular viability. All antibodies and dyes were purchased from Thermo Fisher Scientific. Unstained and single-color compensation controls were used for all flow cytometric experiments, with a minimum of 30,000 events collected/sample. All acquisitions were performed using a S3 Cell Sorter (Bio-Rad) or a BD FACSMelody™ (BD biosciences), and FlowJo (version 10.5.3) was used for data analysis.

### Quantitative PCR (qPCR)

Total RNA was extracted from cells using Trizol reagent (Thermo Fisher Scientific) and converted to cDNA using iSCRIPT cDNA synthesis kit (Bio-Rad). All assays were performed using iQ SYBR Green Supermix (Bio-Rad). An ABI 7500 Real-Time PCR (Applied Biosystems) was used and threshold cycle values that were ≥35 cycles were excluded from the results. Primers were designed using BLAST and purchased from IDT with the following sequences: mouse *β-Actin* forward 5’-AGAGGGAAATCGTGCGTGAC-3’ and reverse 5’-CAATAGTGATGATGACCTGGCCGT-3’, *Myeloperoxidase* (*Mpo)* forward 5’-ACCTACCCCAGTACCGATCC-3’ and reverse 5’-AACTCTCCAGCTGGCAAAAA-3’, *NADPH oxidase* (*Nox-2*, *gp91^phox^, Cybb*) forward 5’-ACTCCTTGGAGCACTGG-3’ and reverse 5’-GTTCCTGTCCAGTTGTCTTCG-3’, and *Il-1β* forward 5’-CCAAAGAAGAAGATGGAAAAGCG-3’ and reverse 5’-GGTGCTGATGTACCAGTTGGG-3’.

### Mice and Hindlimb Unloading

All mice handling and experiments were performed according to the pre-approved NASA Ames Institutional Animal Care and Use Committee (IACUC). Mice were generated for experiments by breeding male, hemizyogous *mCAT* mice [male B6.Cg-Tg (CAG-OTC/CAT) 4033Prab/J strain] (52, 53) with female *wildtype* (*Wt)* mice (*C57BL/6NJ*) (Jackson Laboratories, Bar Harbor, ME). *C57BL/6NJ* *Wt* mice were used as controls. DNA was purified from tail snips using RedExtract-N-Amp (Sigma, St. Louis, MO) followed by genotyping using forward 5’-CTGAGGATCCTGTTAAACAATGC-3’ and reverse 5’-CTATCTGTTCAACCTCAGCAAAG-3’ (54) primers for the *mCAT* gene. For HU experiments, female mice were acclimated to their assigned cages three days prior to the onset of HU. Animals were 16-weeks of age at the beginning of HU. For the 14-day HU study, *C57BL/6NJ* *Wt* female mice were assigned to one of two treatments: normally loaded (NL) controls, singly housed in standard vivarium cages or HU. For the 30-day HU experiment, mice were assigned to one of four groups: *Wt*/NL, *Wt*/HU, *mCAT*/NL, or *mCAT*/HU. In both 14- and 30-day HU studies, mice were housed under 12 h light and 12 h dark cycle conditions and provided cotton nestlets (Ancare, NES3600) as enrichment. Nestlets were refreshed daily. Ambient temperature ranged from 23.3 to 25.6°C. Body weights were monitored every 2–3 days throughout the experiment. Blood draws were performed at euthanasia on days 14 or 30 (32).

### Statistical Analyses

Data were compared with either paired or unpaired, nonparametric or parametric analyses, or with one- or two-way ANOVA using GraphPad Prism software (version 6.0). A *p* < 0.05 was considered statistically significant. All statistical analyses were supported by a trained statistician.

## Results

### Spaceflight Elevates NLR and GLR

**Figure 1:**
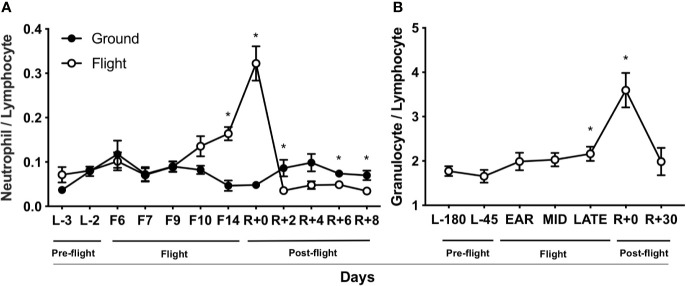
Spaceflight elevates NLR and GLR. **(A)** Rodent NLR from Space Life Sciences (SLS)-2 mission (47, 48) (n = 5–15). **(B)** Human GLR from published data (29) (n = 23). L, launch; F, flight; R, return on Earth denoted in days. “Ear”ly, day 14 in-flight; “mid,” days 60–120 in-flight; and “late,” day 180. A non-parametric, unpaired Mann-Whitney test compared ground controls with in-flight samples at each timepoint in rodent data set and a parametric, paired Student’s *t*-test compared to L-180 days was performed in human data set, a * indicates p < 0.05. Error bars denote standard error of mean.

Peripheral WBC data from previously space-flown rodent and astronaut experiments were re-analyzed to determine the contribution of spaceflight to NLR and GLR immune profile shifts. In rodents (47, 48), spaceflight increased NLR after 14 days in-flight and immediately post-landing (**Figure 1A**). Later post-flight (2–8 days after landing, R+2 to R+8), NLR decreased relative to flight (F14) and landing (R+0) values, suggesting a re-adaptation response to Earth’s 1 gravity (1 g) (**Figure 1A**). Retrospective GLR data were not recorded for this spaceflight mission. Human WBC data (29) were re-analyzed and GLR was elevated after 180 days on-orbit (late) and in samples collected within 2–3 hours post-landing (R+0) (**Figure 1B**). Later post-flight (30-day, R+30), GLR recovered to pre-flight baseline levels (L-180). Retrospective NLR data were not recorded for this mission. Thus, a progressive increase in NLR and GLR occurred in-flight and immediately post-landing in rodents and humans, suggesting NLR may be a useful biomarker to monitor astronaut immune status.

### HARV-RWV sµg Elevates NLR in Human Leukocytes *In Vitro*

**Figure 2:**
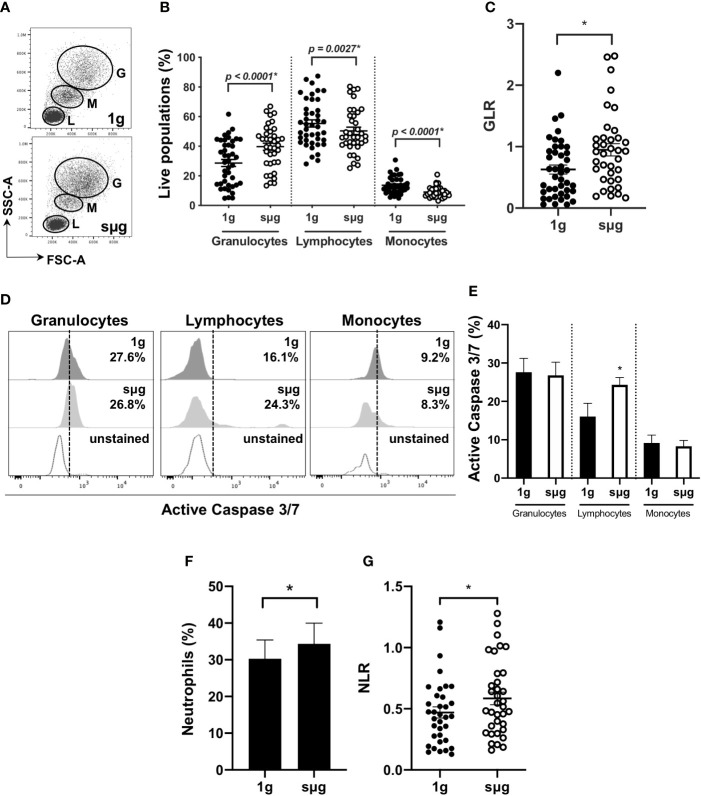
HARV-RWV sµg elevates GLR and NLR. **(A)** Flow scatter plot: G, Granulocyte; M, Monocyte; L, Lymphocytes. **(B)** Percent (%) live population of each cell type (n = 37–42). **(C)** GLR based on % population of each cell type (n = 37–42). **(D)** Representative flow histogram plots of active Caspase 3/7 within each population type. **(E)** Percent (%) active Caspase 3/7 fluorescence within all events per leukocyte population (n = 10). **(F)** Bar graph of neutrophils (CD66b^+^CD16^+^) within WBC post-20 h incubation at 1 g and sµg (n = 28). **(G)** Total NLR (n = 28). All experiments were repeated at least twice. A non-parametric, Wilcoxon matched pairs signed rank test compared to 1 g was performed for sµg leukocyte differential analyses and GLR/NLR determination. A * indicates p < 0.05 and error bars denote standard error of mean.

Due to the constraints of conducting spaceflight experiments, we further confirmed these results using an *in vitro* sµg model. For this, human WBC were cultured in HARV-RWV sµg for 20 h. Flow cytometry showed sµg increased granulocyte percentage (%) and absolute counts, reduced lymphocyte and monocyte %, and although not statistically significant, a reduced trend in absolute counts (forward scatter area, FSC-A *versus* side scatter area, SSC-A) (**Figures 2A, B**, and **Figure S1A**), and increased GLR (**Figure 2C**). To determine if altered GLR was due to elevated survival of granulocytes or increased death of lymphocytes, active Caspase 3/7 staining was performed, which indicated elevated lymphocyte apoptosis in sµg **(** **Figures 2D, E** **)**. To characterize human neutrophil populations within the WBC pool following sµg, cell surface markers CD66b^+^ and CD16^+^ were used (55–58), which displayed elevated neutrophils (**Figure 2F**) and elevated NLR (**Figure 2G**). Collectively, these findings confirm the utility of NLR as a biomarker to monitor astronaut immune status.

### HARV-RWV sµg Elevates ROS and Activates Neutrophils, While the Antioxidant *N*-Acetyl Cysteine Ameliorates This Effect

**Figure 3:**
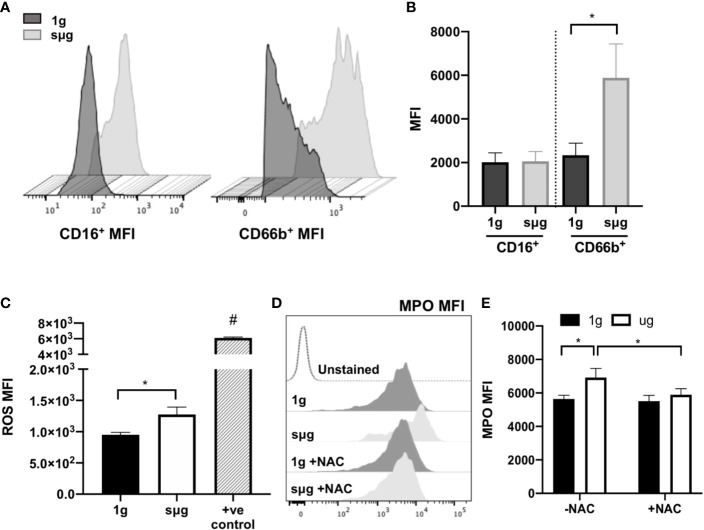
HARV-RWV sµg activates neutrophils to produce ROS and MPO, while antioxidant treatment ameliorates this effect. **(A)** Representative CD16^+^ and CD66b^+^ median fluorescence intensity (MFI) histograms. **(B)** Median fluorescence intensity (MFI) cell surface expression of CD16^+^ and CD66b^+^ per granulocyte (n = 32). **(C)** CellROX measurement of mean fluorescence intensity (MFI) of ROS per granulocyte (n = 10). Positive controls (+ve control) included a 30-min incubation with the ROS-inducer *tert*-Butyl hydroperoxide (TBHP, 400 µM) (n = 2). **(D)** Representative flow histogram plot of MPO mean fluorescence intensity (MFI). **(E)** MPO MFI per neutrophil (CD66b^+^CD16^+^) in the presence or absence of the antioxidant, *N*-acetyl cysteine (NAC, 1 mM, n = 10–24). All experiments were repeated at least twice. A non-parametric, Wilcoxon matched pairs signed rank test compared to 1g or control groups, a * indicates p < 0.05, a # indicates the positive control with a p<0.05 compared to 1g. Error bars denote standard error of mean.

Elevated percentage and absolute count of granulocytes within WBC (**Figures 2A, B**, and **Figure S1A**) were observed in sµg, with no difference in apoptosis (**Figures 2D, E**). Elevated percentage of neutrophils within WBC (**Figure 2F**) were also observed in sµg. Since mature granulocytes, including neutrophils, in blood are non-proliferating, terminally differentiated cells, elevated percentages may be due to differential light scatter properties, indicative of cellular activation. CD66b not only serves as a marker for human neutrophils, but its cell surface expression level per cell is also elevated in activated neutrophils (58, 59). Therefore, we sought to determine if sµg can activate neutrophils. We found sµg resulted in elevated cell surface receptor median fluorescence intensity (MFI) expression of CD66b per granulocyte, with no difference in CD16 MFI (**Figures 3A, B**), suggesting neutrophil activation during sµg. We further confirmed neutrophil activation by uncovering elevated cell surface receptor CD11b median fluorescence intensity (MFI) within CD16^+^CD66b^+^ granulocytes (**Figures S1B, C**). Activated neutrophils also express elevated reactive oxygen species (ROS) and myeloperoxidase (MPO) during the oxidative burst response (11, 60–62). Furthermore, spaceflight and analog models on Earth (4–9, 63) can promote redox imbalance, triggering cellular damage and persistent inflammation (12). We found ROS (*via* mean fluorescence intensity, or MFI) per granulocyte and MPO (mean fluorescence intensity, MFI) per neutrophil, were both elevated in sµg, collectively suggesting sµg caused neutrophil activation (**Figures 3C, E**).

*N*-acetyl cysteine (NAC) is an antioxidant that scavenges free radicals, promotes glutathione biosynthesis, and decreases mitochondrial membrane depolarization (64). To assess the effects of NAC on neutrophil activation we cultured WBCs in the presence or absence of NAC (1 mM) under sµg for 20 h. The results showed reduced expression of MPO in the presence of NAC (**Figures 3D, E** **)**, suggesting antioxidant treatment ameliorates sµg induced neutrophil activation, thus serving as a promising countermeasure to suppress spaceflight-induced inflammation.

### Hindlimb Unloading (14-day) sμg Increases Circulating Blood Neutrophils and Elevates NLR *In Vivo*

**Figure 4:**
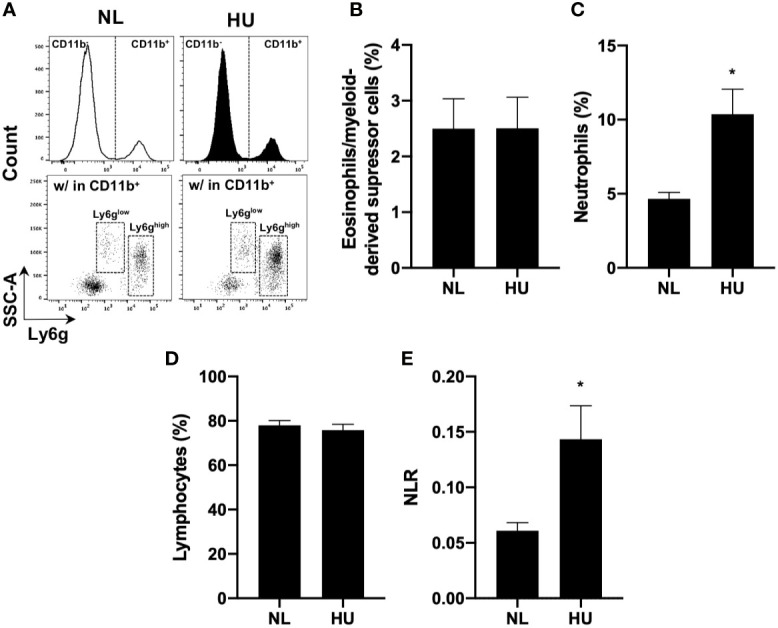
14-day HU sµg increases number of circulating blood neutrophils and elevates NLR. Blood from HU and NL (14-day) *Wt* mice. **(A)** Representative flow cytometric gating scheme for Ly6g^low^ (eosinophils/myeloid-derived suppressor cells) (65) and Ly6g^high^ (neutrophils) within CD11b^+^/CD45^+^ myeloid cells and CD11b^-^/CD45^+^ lymphocytes. % of eosinophils/myeloid-derived suppressor cells **(B)**, neutrophils **(C),** and lymphocytes **(D)**. **(E)** NLR deduced from neutrophils (Ly6g^high^ CD11b^+^/CD45^+^ events) to lymphocytes (CD11b^-^/CD45^+^ events) (n = 7). A non-parametric, unpaired Mann-Whitney test compared to NL controls was performed, a * indicates p < 0.05. Error bars denote standard error of mean.

To test the effects of sµg on immunity in an *in vivo* model (32), blood was collected from *wildtype* HU (*Wt*/HU) mice following 14 days of HU. Cells were immunoprofiled to determine neutrophil counts and NLR in circulating blood. Ly6g is a ubiquitous cell surface marker in mice used to distinguish eosinophils/myeloid-derived suppressor cells (Ly6g^low^) from neutrophils (Ly6g^high^) (65) (**Figure 4A** **)**. Compared to normally loaded (NL) controls, no difference was observed in eosinophil/myeloid-derived suppressor cell % (**Figure 4B**) and absolute counts (**Figure S2A**) populations, while increased % (**Figure 4C**) and absolute counts (**Figure S2B**) of neutrophils were observed in HU. No difference in lymphocyte % (**Figure 4D**) or absolute counts (**Figure S2C**) were noted in the *in vivo* HU model, in contrast with the reduced lymphocytes % observed *in vitro* HARV-RWV sµg-treated leukocytes (**Figure 2B**) with increased apoptosis of lymphocytes. No significant differences were observed with monocyte % or absolute counts (**Figures S2D, E**). However, significantly elevated NLR was observed following 14 days of HU (**Figure 4E**). Therefore, HU (14-day) displayed elevated NLR values as observed previously with rodents and humans in spaceflight and sµg experiments, confirming elevated NLR in multiple reduced gravity models, albeit produced *via* potentially different mechanisms.

### Prolonged Hindlimb Unloading (30-day) sµg Results in Elevated Blood Neutrophil Persistence and NLR, While This Effect is Mitigated in *mCAT* Mice

**Figure 5:**
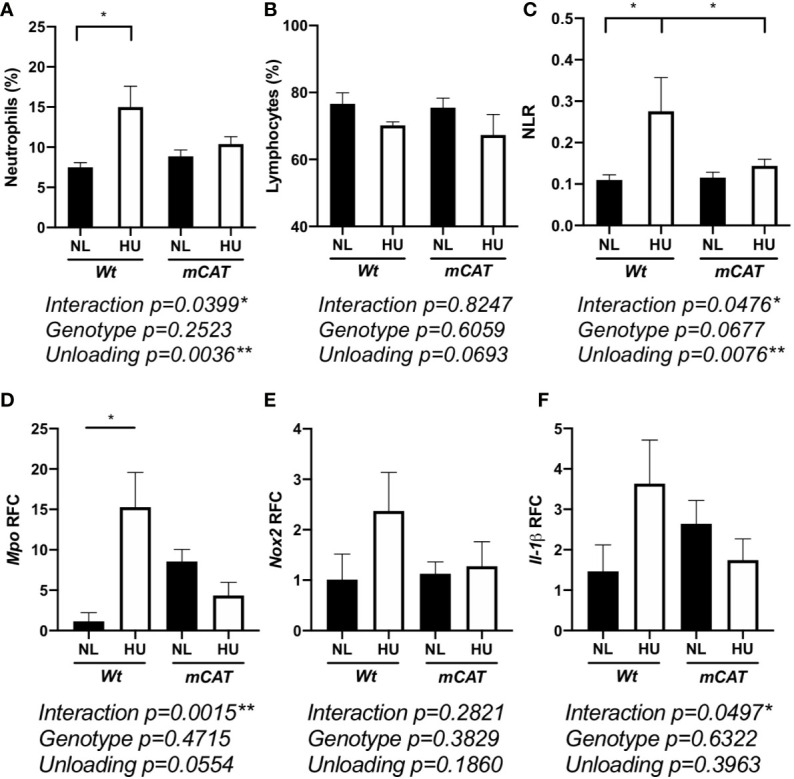
30-day HU sµg results in elevated blood neutrophil persistence and NLR with this effect mitigated in *mCAT* mice. Blood from HU (30-day) *Wt* and *mCAT* mice. % neutrophils (Ly6g^high^ CD11b^+^/CD45^+^ events) **(A)**, lymphocytes (CD11b^-^/CD45^+^) **(B)**, and NLR **(C)** from NL and HU mice (n = 5–8). qPCR relative fold change (RFC) of *Mpo* ***(*D*)***, *Nox-2* ***(*E*)***, and *Il-1β* **(F)** in blood collected from *Wt* and *mCAT*, NL and HU mice (30-day, n = 3–8). A two-way ANOVA and a non-parametric, Dunn’s multiple comparisons test was performed between groups, a * indicates p < 0.05. Error bars denote standard error of mean.

Elevated neutrophil numbers and persistence in blood circulation result in tissue damage and impaired immune responses (66). HU also induces redox imbalance (8). Therefore, we compared the effects of prolonged HU (30-day) in *Wt* mice with transgenic mice expressing human mitochondrial *catalase (mCAT)* (54). Catalase is an antioxidant enzyme that converts reactive hydrogen peroxide into non-reactive water and oxygen, a cellular antioxidant mechanism that restores redox balance. Comparable to 14-day HU, 30-day HU in *Wt* mice resulted in elevated neutrophils, no significant difference in lymphocytes, and an elevated NLR, while these results were mitigated in *mCAT*/HU mice (**Figures 5A–C** and **Figure S3**). Oxidative stress and inflammatory gene expression in *Wt* and *mCAT* mice were assessed by qPCR. Compared to *Wt*/NL controls, *Mpo* (p = 0.0240*) was increased in *Wt/*HU (**Figure 5D**), while a non-significant elevation in *NADPH oxidase-2* (*Nox-2*, *p = 0.6411*) and *Il-1β* (*p = 0.1349*) were also observed (**Figures 5E, F**). On the other hand, *mCAT*/HU mice partially mitigated some of these effects (**Figures 5D–F**). Collectively, prolonged HU (30-days) induced persistent NLR, oxidative stress and marginal inflammation, while *catalase* overexpression mitigated some of these outcomes.

Collectively, our findings demonstrated that, through potentially different mechanisms, both spaceflight and multiple sµg models elevated NLR, ROS, and MPO inflammation, while antioxidants mitigated some of these outcomes. Therefore, elevated NLR may be a suitable prognostic biomarker to monitor astronaut immune status and inflammation during long-duration missions.

## Discussion

Spaceflight causes immune dysfunction that can lead to health risks for astronauts. Health risks that arise from immune dysfunction are complex and include an inability to defend against pathogens, altered tolerance to self-antigens resulting in potential autoimmunity development, chronic inflammation, and immune senescence. Therefore, mitigation during spaceflight will likely require selective targeting of specific immune cell types and/or developmental stages. Elevated NLR may be a prospective biomarker candidate to identify immune deviations that can cause disease. Currently, elevated NLR is used as a clinical biomarker to detect sub-clinical inflammation in humans and predicts poor prognosis in cancer (18). In this study, a spike in GLR in humans and NLR in rodents was observed at landing and elevated GLR and NLR were observed during spaceflight (**Figure 1**). In humans, GLR was elevated at 180-days in-flight, suggesting prolonged exposure to spaceflight caused leukocyte differential changes. This change in immune differentials may be in response to elevated inflammation experienced in-flight (4, 6, 12), since elevated, chronic inflammation is often coupled with immune dysfunction and disease development (13, 19, 20, 67–70). Therefore, monitoring distinct biomarkers, such as elevated NLR, can determine when countermeasures can intervene to avert immune dysfunction, promote immune recovery, and prevent disease development.

Elevated numbers of granulocytes and neutrophils were observed *in vitro* following 20 h of HARV-RWV modeled microgravity in human peripheral blood (**Figure 2** and **Figure S1**). However, since mature neutrophils are terminally differentiated in blood circulation, i.e., banded or segmented neutrophils, we estimate that this increased percentage is due to increased scatter properties indicative of cellular activation. Indeed, elevated cell surface expression of CD66b and CD11b per granulocyte, which are activation markers for neutrophils (58, 59), were increased in sµg (**Figure 3** and **Figure S1**). Further studies revealed elevated ROS and MPO expression (**Figure 3**), confirming HARV-RWV sµg resulted in granulocytes, and in particular, neutrophil activation. Physiological effects of elevated active granulocytes or neutrophils in circulation intensifies sterile inflammation (70–72), promotes edema, and non-specific tissue damage (73), and can threaten astronaut health if not adequately controlled. Interestingly, MPO gene expression (**Figure 5D**) was elevated in HU, suggesting immature neutrophil entry into blood circulation, compared to neutrophil activation observed *in vitro* in the HARV-RWV. MPO gene synthesis only occurs in bone marrow early in neutrophil development, i.e. immature neutrophils (myeloblasts, promyelocytes, and myelocytes). MPO gene expression ends once neutrophils differentiate into metamyelocytes (74) and synthesized MPO protein is packaged into granules released during neutrophil activation (75). Typically, immature neutrophils are not released into blood circulation unless the body is in a diseased or inflammatory state (74, 75). However, since MPO gene expression was elevated in HU mice blood, this suggests the potential for myelocyte/immature neutrophil infiltration and may also serve as a biomarker for elevated inflammation during spaceflight. To our knowledge no measurements have been recorded for elevated immature neutrophils in blood circulation in-flight; however, elevated neutrophils were identified in blood from 9 of 16 astronauts at landing (6, 76, 77). As compared to the rodent spaceflight results (in-flight day 14), elevated neutrophils were also observed at days 14 and 30 of HU in mice (**Figures 4C** and **5A**), suggesting the physical effects of fluid-shifting experienced during spaceflight and HU may stimulate the release of neutrophils into circulation; however this requires further investigation.

Indeed, elevated MPO during sµg may contribute to immune dysfunction. MPO catalyzes hydrogen peroxide into reactive intermediates that can damage proteins, lipids, and DNA (67). Excess MPO impairs phagocytic function (67–69) and triggers neutrophil degranulation, causing inflammatory tissue damage (78) in cardiovascular disease (62, 67, 79). Pathologically this is relevant during spaceflight, as cardiovascular disease is a prominent risk factor associated with returned astronauts (80). Furthermore, elevated NLR is currently used as a predictor of cardiovascular disease risk on Earth (81), thereby highlighting the clinical relevance of monitoring NLR during spaceflight. Neutrophil oxidative burst responses and elevated ROS can induce cellular death (82), including lymphocyte apoptosis (83), and suppression of T lymphocyte function (84). HARV-RWV sµg induced ROS in granulocytes, indicating HARV-RWV sµg activated granulocytes and triggered the oxidative burst response. Thus HARV-RWV sµg can serve as a valuable model to study ROS-induced inflammation (72, 85). Indeed, redox imbalance occurs in humans and cell cultures exposed to spaceflight (4–6) and *in vitro* ground-based sµg models (8, 9, 86). The cause of elevated ROS in sµg may be due to: (1) cell death factors or other unknown stimulators of the oxidative burst response, (2) a mechanosensitive stress receptor in phagocytes that triggers redox imbalance (87), and/or (3) a combination of these effects. In our study, HARV-RWV sµg induced active Caspase 3/7 expression in lymphocyte populations (**Figure 2D**), indicating lymphocyte apoptosis and shifting of immune differentials to favor higher NLR and GLR. Further analyses of our *in vivo* HU studies revealed no difference in lymphocyte percent or absolute count populations (**Figures 4** and **5**, and **Figures S2** and **3**) compared to HARV-RWV sµg studies, suggesting elevated lymphocyte recovery or unknown ROS-quenching mechanisms that limit lymphocyte apoptosis, both of which require further studies.

Therefore, the two sµg models, HARV-RWV and HU, displayed different mechanisms towards generating an elevated NLR. The HARV-RWV microgravity model appears to display robust lymphocyte turnover, i.e. elevated lymphocyte apoptosis, and most likely immune function that may differ from astronauts in-flight. However, this does not rule out that lymphocyte apoptosis does not occur *in vivo* HU, as turnover of lymphocytes to replace loss most likely occurs, albeit apoptosis may occur at a slower rate than *in vitro* HARV-RWV. Furthermore, ROS concentration within each sµg model may be drastically different. For example, ROS levels in HARV-RWV may be much higher in the absence of *in vivo* ROS quenchers compared to 14- or 30-day HU, which would affect the rate of lymphocyte apoptosis (88, 89). Indeed, concentration and exposure time of ROS determines cellular responses. Homeostatic levels of ROS can promote cell survival, while elevated ROS (oxidative stress) can induce cellular death (82). In line with this, the timeline of measurements of lymphocyte counts (20 h HARV-RWV *versus* 14- and 30-days HU) differ between the two models; therefore direct comparisons cannot be assumed. Finally, the HARV-RWV model cultured human blood samples, which have different leukocyte percentages compared to mice leukocytes in the HU model; therefore the kinetics of apoptosis across the two microgravity models would also be affected. Crucian et al. showed there is an elevation of granulocytes in blood circulation, while no differences are observed in lymphocyte absolute counts, suggesting lymphocytes may not undergo apoptosis in spaceflight; rather there may be release of more granulocytes into blood circulation (29). Controversially however, multiple reports have indicated lymphocytes and lymphocyte-like cell lines undergo apoptosis during spaceflight/microgravity conditions (36–42), albeit measurements were either reported post-landing or from *ex vivo* cell cultures in flight. In fact, the role of apoptosis in lymphocyte depression (ROALD) experiment that was part of the BIO-4 mission and comprised of ESA, Energia, and NASA agencies, was performed with the goal to understand how microgravity affects lymphocyte apoptosis. The results showed after 48 h on-board the ISS *ex vivo* cultures of lymphocytes displayed increased DNA fragmentation, PARP protein expression, and elevated p53 expression, compared to ground controls (36). Due to this, we believe the *in vivo* HU microgravity model, although having its own limitations, may be a better representative ground-based model for spaceflight. Nonetheless, additional studies are required to better understand the degree of lymphocyte turnover during *in vivo* HU that is comparable to spaceflight.

Monocytes were significantly reduced following HARV-RWV (**Figure 2B**); however no differences were observed in HU mice (**Figures S2D, E**), further indicating the variability between the two simulated microgravity models. Yet, inconsistency with these cell types in terms of population differentials have also been noted across spaceflight literature (77, 90, 91), which may be a factor of sampling timepoints. However, consensus suggests phagocytic function of monocytes following spaceflight is impaired (92, 93). Phagocytic impairment of monocytes can directly affect clearance of neutrophils from circulation, inflammation resolution (94), and can impact NLR. Therefore, further research into the function and distribution of monocytes following simulated microgravity are currently underway.

Transgenic mice expressing the human antioxidant gene *catalase* reversed HU-induced elevation of NLR and dampened inflammatory gene expression (**Figure 5**), suggesting redox imbalance caused leukocyte differential changes. In line with this, mice deficient in apolipoprotein E (ApoE), a protein with antioxidant activity, display elevated ROS expression and activated neutrophils (95). In our study, HARV-RWV sµg of human leukocytes induced neutrophil activation that was reversed in the presence of the antioxidant NAC (**Figure 3D**), further suggesting antioxidants can suppress inflammation. Indeed, *in vivo* NAC treatment successfully ameliorated acetic acid-induced colitis by reversing pro-inflammatory mediators TNF-α, IL-6, and MPO in rats (96). Collectively, these results indicate antioxidants as viable countermeasures to regulate spaceflight-induced inflammation and immune dysfunction.

Clinically, elevated NLR (>3.53) (27) is a prognostic indicator for cancer development, cardiovascular disease, inflammation, and infectious conditions (18, 21–23, 25), but no NLR standard has been established for astronaut immunity. Our results revealed elevated GLR in astronauts at landing (GLR = 3.6, **Figure 1B**), compared to clinically relevant GLR (>2.24) (97), may result in biological significance for astronaut health. Although restoration to normal GLR occurred at landing on Earth, landing on the lunar surface and/or Mars, where gravity is less than Earth’s, may pose a significant risk to astronaut immune recovery. Therefore, monitoring and developing countermeasures to mitigate elevated NLR, GLR, and inflammatory neutrophil phenotypes for future long-duration and long-distance space travel are essential for mission success.

In summary, we identified increased GLR and NLR in both human and rodent spaceflight samples and ground simulations of microgravity. Our results *in vitro* indicated that leukocytes shift in favor of elevated activated inflammatory neutrophils, which may amplify disease development *in vivo*. Further, antioxidants may be useful countermeasures to ameliorate these outcomes in sµg, as NAC treatment inhibited activated inflammatory human neutrophils in HARV-RWV and *catalase* partially mitigated elevated NLR in HU mice. Based on these findings, we suggest monitoring both in-flight and landing NLR to assess astronaut immune status. We further advocate the investigation of antioxidants as future countermeasures to mitigate immune deviations, including elevated NLR and inflammation, to safeguard astronaut health on future missions.

## Data Availability Statement

The data analyzed in this study are subject to the following licenses/restrictions: NASA Life Sciences Data Archive (LSDA) is an active repository for astronaut health datasets. Requests for dataset access is required. Requests to access these datasets should be directed to https://lsda.jsc.nasa.gov.

## Ethics Statement

The studies involving human participants were reviewed and approved by NASA Ames Institutional Review Board (IRB, 201791646CTO-02, HR-357 and HR-358) with informed consent from each blood donor. Astronaut and rodent CBC data sets were approved for use by the electronic (e)IRB/Life Sciences Data Archive (LSDA) advisory board (#11028). The patients/participants provided their written informed consent to participate in this study. The animal study was reviewed and approved by NASA Ames Research Center Institutional Animal Care and Use Committee (IACUC, NAS-17-001-Y2).

## Author Contributions

AMP conceived/performed majority of the experiments and wrote/prepared the manuscript. SDM performed experiments and contributed to manuscript preparation. EC provided human blood samples and edited the manuscript. A-SS, CGTT, and RKG performed the HU animal experiments, provided mouse blood samples, and edited the manuscript. SA assisted with statistical testing and edited the manuscript. RG, BEC, and SB edited the manuscript and provided intellectual advice. SB provided funding for the study. BEC provided human LSDA-sourced data sets and edited the manuscript. All authors contributed to the article and approved the submitted version.

## Funding

This work was supported in part by Universities Space Research Association (USRA) and NASA’s Space Biology Program post-doctoral fellowship (to AMP) and NASA’s Space Biology Grant # NNX15AB42G (to SB).

## Conflict of Interest

The authors declare that the research was conducted in the absence of any commercial or financial relationships that could be construed as a potential conflict of interest.
