# Supplementary material for: Neutrophil-to-Lymphocyte Ratio: A Biomarker to Monitor the Immune Status of Astronauts
Source: Front Immunol. 2020 Nov 2;11:564950. doi: 10.3389/fimmu.2020.564950 (PMC7667275; doi:10.3389/fimmu.2020.564950)
Supplement: Supplementary file 1 [file DataSheet_1.pdf]

Supplementary Materials

Neutrophil-to-Lymphocyte ratio: A biomarker to monitor the immune status of astronauts

Amber M. Paul,[1,2] Siddhita D. Mhatre,[1,3] Egle Cekanaviciute,[1,2] Ann-Sofie Schreurs,[1,2] Candice G.T. Tahimic,[1,3] Ruth K. Globus,[1] Sulekha Anand,[4] Brian E. Crucian,[5] and Sharmila Bhattacharya[1*]

[1]Space Biosciences Division, NASA Ames Research Center, Moffett Field, CA, USA
[2]Universities Space Research Association, Columbia, MD, USA
[3]KBR, Houston, TX, USA
[4]Department of Biological Sciences, San Jose State University, San Jose, CA, USA
[5]Biomedical Research and Environmental Sciences Division, NASA Johnson Science Center, Houston, TX, USA

*** Correspondence:**
Dr. Sharmila Bhattacharya, Space Biosciences Research Branch, NASA Ames Research Center, Moffett Field, CA, USA, 94035; Telephone: 650-604-153.
Email: Sharmila.Bhattacharya@nasa.gov

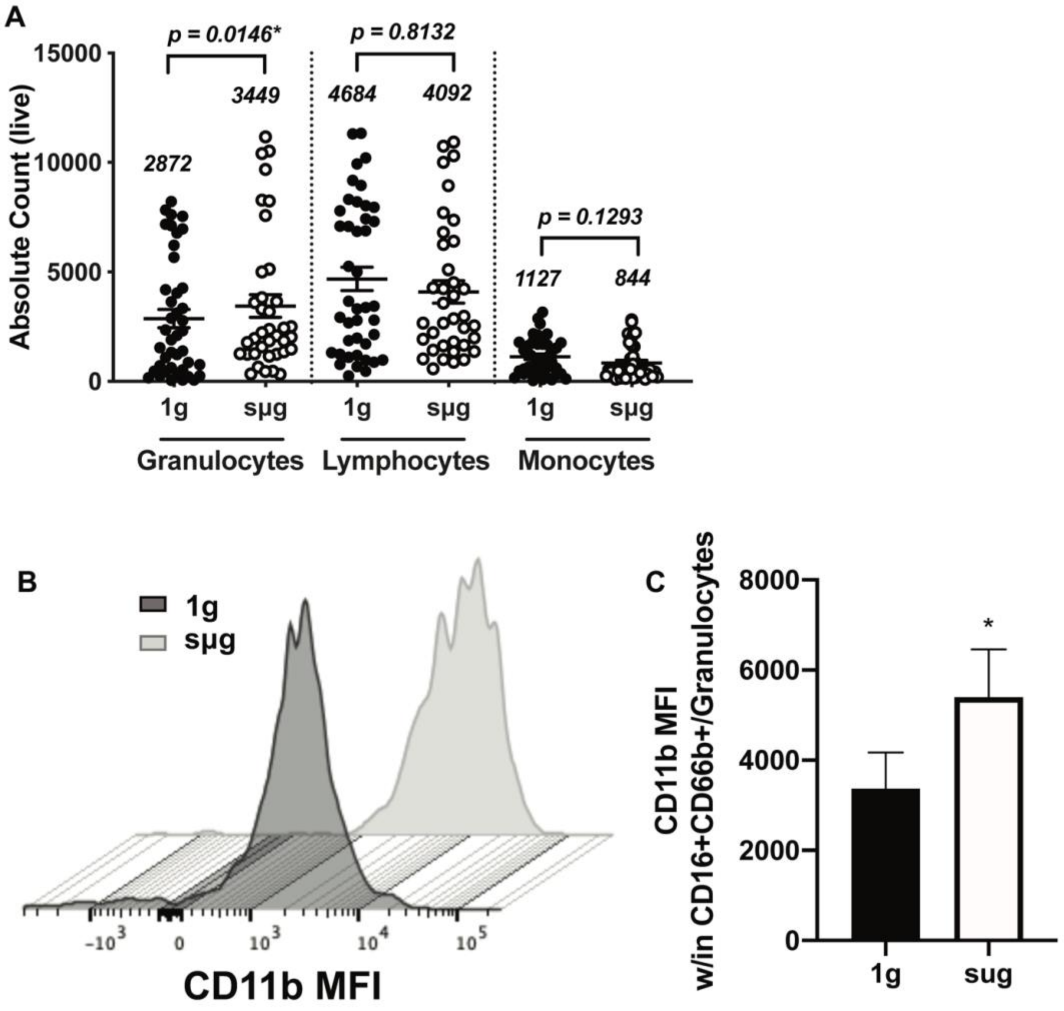

Supplemental Figure 1. HARV-RWV sμg elevated absolute counts of activated granulocytes. (A) Absolute count of live populations (Sytox negative) of each cell type (n = 37-42). **(B)** Representative $CD11b^+$ median fluorescence intensity (MFI) histograms. **(C)** Median fluorescence intensity (MFI) cell surface expression of $CD11b^+$ per $CD16^+CD66^+$ granulocyte (n= 32). All experiments were repeated at least twice. A non-parametric, Wilcoxon matched pairs signed rank test compared to 1g was performed. A * indicates $p<0.05$ and error bars denote standard error of means.

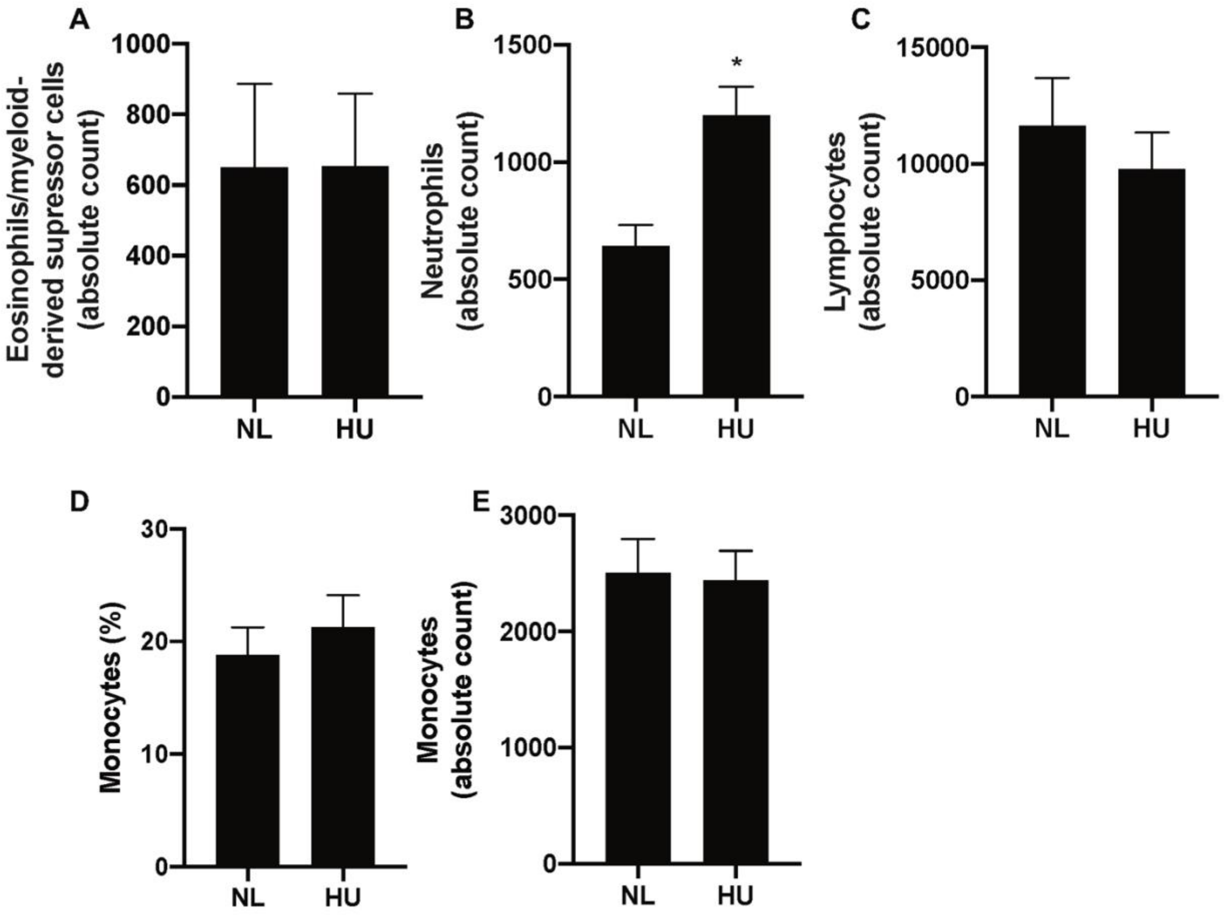

Supplemental Figure 2. 14-day HU sµg increases number of absolute counts of neutrophils. Blood from HU and NL (14-day) *Wt* mice. Absolute count of eosinophils/myeloid-derived suppressor cells **(A)**, neutrophils **(B)**, and lymphocytes **(C)**. Percentage (%) **(D)** and absolute count **(E)** of CD11b$^+$/CD45$^+$ monocytes (n=7). A non-parametric, unpaired Mann-Whitney test compared to NL controls was performed, a * indicates $p<0.05$. Error bars denote standard error of means.

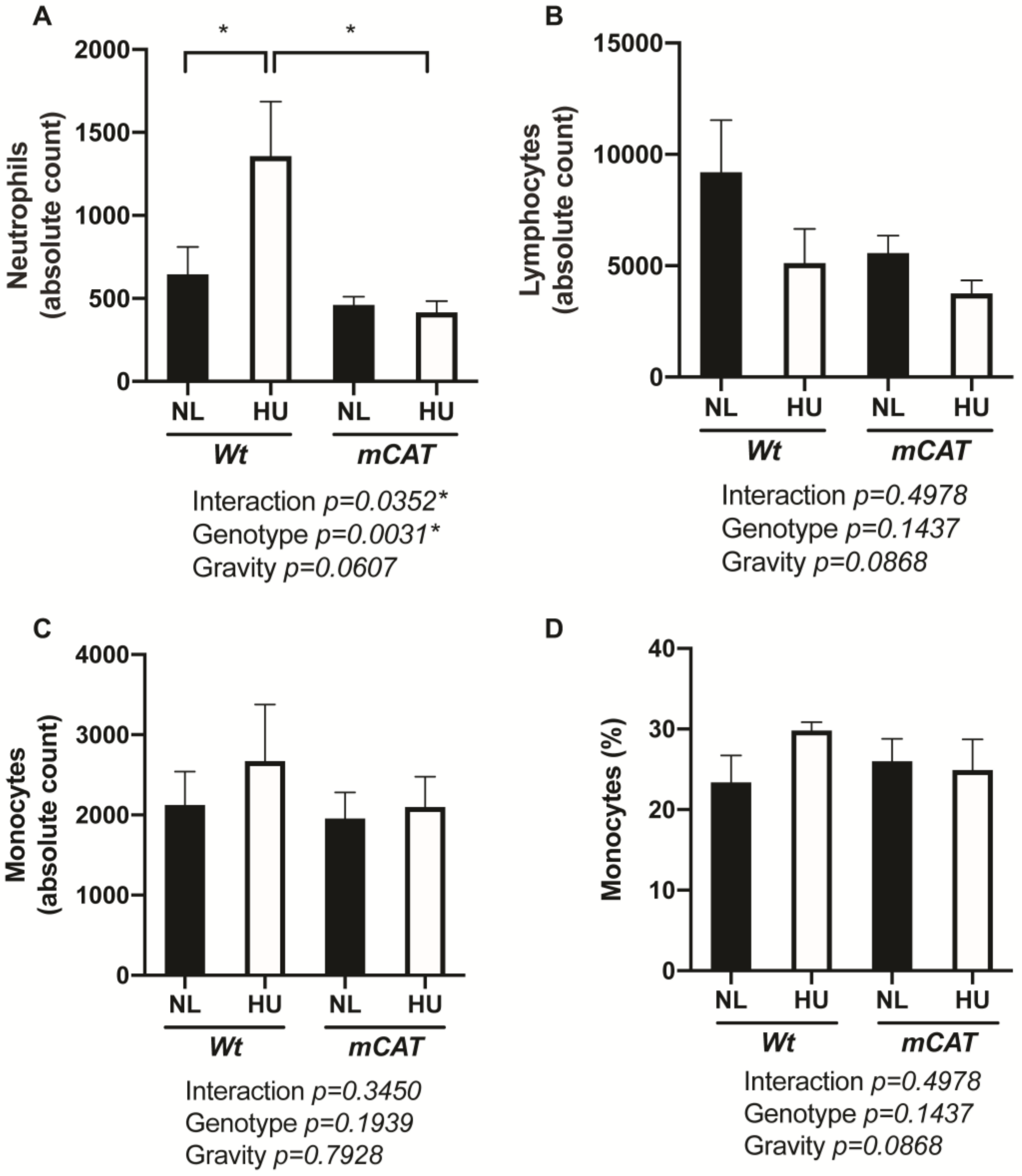

Supplemental Figure 3. 30-day HU elevates blood neutrophil absolute counts and is mitigated in *mCAT* mice. Blood from HU (30-day) *Wt* and *mCAT* mice. Absolute counts of neutrophils **(**$Ly6g^{high}$ $CD11b^{+}/CD45^{+}$ events) **(A)**, lymphocytes ($CD11b^{-}/CD45^{+}$) **(B)**, monocytes ($CD11b^{+}/CD45^{+}$) **(C)** , and percentage (%) of monocytes **(D)** (n = 5-8). A two-way ANOVA and a non-parametric, Dunn's multiple comparisons test was performed between groups, a * indicates p<0.05. Error bars denote standard error of means.
